# Supplementary material for: The interactome of intact mitochondria by cross-linking mass spectrometry provides evidence for coexisting respiratory supercomplexes
Source: Mol Cell Proteomics. 2017 Dec 8;17(2):216–32. doi: 10.1074/mcp.RA117.000470 (PMC5795388; doi:10.1074/mcp.RA117.000470)
Supplement: Supplemental Data [file supp_RA117.000470_133922_0_supp_23978_fzffwf.zip › spectra_annotation/mito_spectra_annotation/88-2-8-1-7-1.pdf]

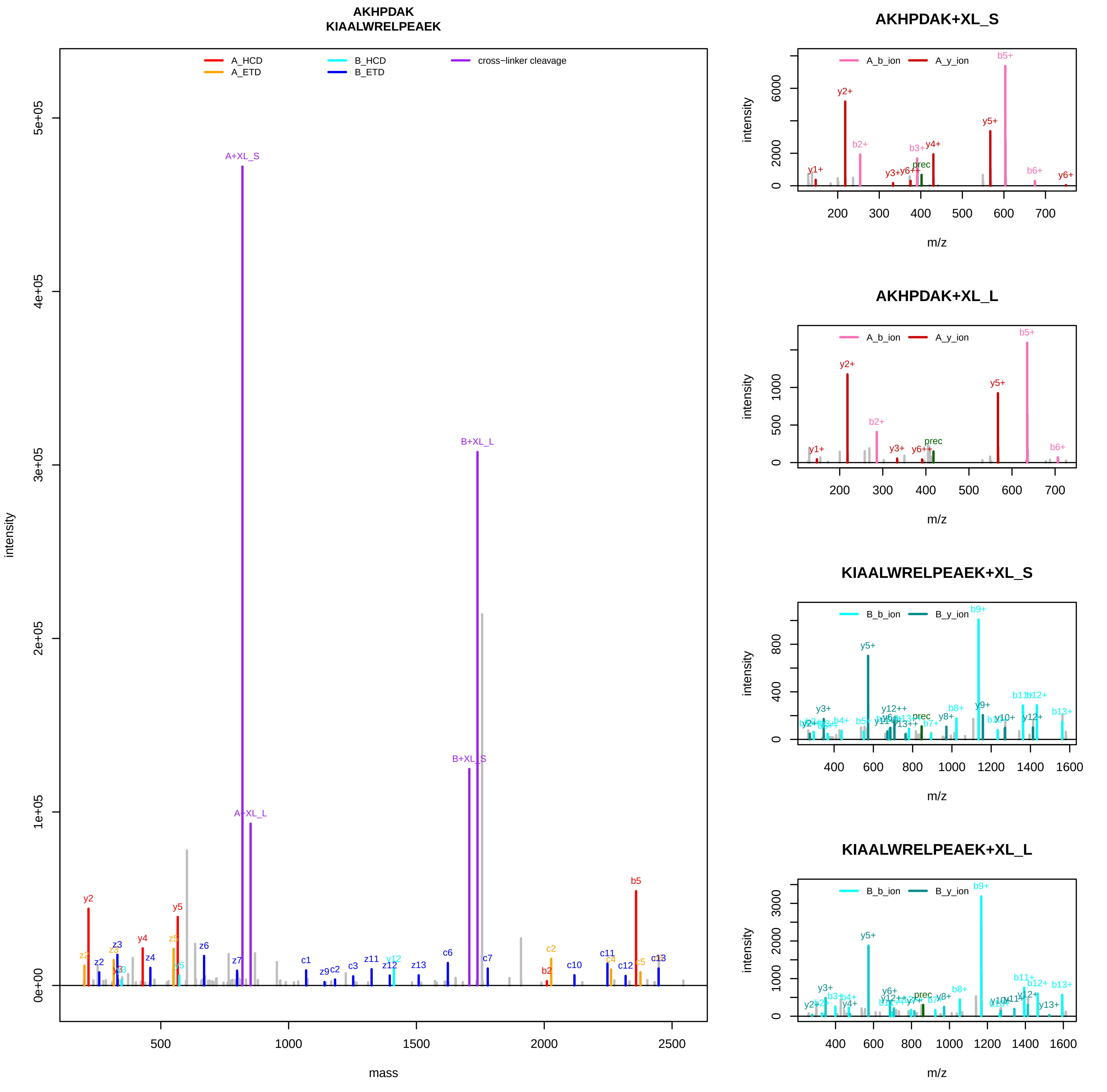

AKHPDAK
KIAALWRELPEAEK
A_HCD
A_ETD
B_HCD
B_ETD
cross−linker cleavage
A+XL_S
A+XL_L
B+XL_L
B+XL_S
y2
y4
y5
b5
b2
z2
z3
z4
z5
z6
z7
z9
z11
z12
z13
c1
c2
c3
c4
c5
c6
c7
c10
c11
c12
c13
y12
intensity
mass
0e+00
1e+05
2e+05
3e+05
4e+05
5e+05
500
1000
1500
2000
2500
AKHPDAK+XL_S
AKHPDAK+XL_L
KIAALWRELPEAEK+XL_S
KIAALWRELPEAEK+XL_L
A_b_ion
A_y_ion
B_b_ion
B_y_ion
y1+
y2+
y3+
y4+
y5+
y6+
y6++
b2+
b3+
b5+
b6+
b9+
b11+
b12+
b13+
y12++
y13++
prec
m/z
